# Genome Sequence of *Porticoccus hydrocarbonoclasticus* Strain MCTG13d, an Obligate Polycyclic Aromatic Hydrocarbon-Degrading Bacterium Associated with Marine Eukaryotic Phytoplankton

**DOI:** 10.1128/genomeA.00672-15

**Published:** 2015-06-18

**Authors:** Tony Gutierrez, William B. Whitman, Marcel Huntemann, Alex Copeland, Amy Chen, Nikos Kyrpides, Victor Markowitz, Manoj Pillay, Natalia Ivanova, Natalia Mikhailova, Galina Ovchinnikova, Evan Andersen, Amrita Pati, Dimitrios Stamatis, T. B. K. Reddy, Chew Yee Ngan, Mansi Chovatia, Chris Daum, Nicole Shapiro, Michael N. Cantor, Tanja Woyke

**Affiliations:** a School of Life Sciences, Heriot-Watt University, Edinburgh, United Kingdom; b Department of Microbiology, University of Georgia, Athens, Georgia, USA; c DOE Joint Genome Institute, Walnut Creek, California, USA

## Abstract

*Porticoccus hydrocarbonoclasticus* strain MCTG13d is a recently discovered bacterium that is associated with marine eukaryotic phytoplankton and that almost exclusively utilizes polycyclic aromatic hydrocarbons (PAHs) as the sole source of carbon and energy. Here, we present the genome sequence of this strain, which is 2,474,654 bp with 2,385 genes and has an average G+C content of 53.1%.

## GENOME ANNOUNCEMENT

*Porticoccus hydrocarbonoclasticus* strain MCTG13d was isolated from a laboratory culture of the marine dinoflagellate *Lingulodinium polyedrum* (CCAP 1121/2) by enrichment with polycyclic aromatic hydrocarbons (PAHs) as the sole carbon source (1). The strain represents a novel species of obligate hydrocarbonoclastic marine bacteria (OHCB) that exhibit a narrow nutritional spectrum, preferring to utilize aliphatic and aromatic hydrocarbons and small organic acids (2). Notably, strain MCTG13d displays versatility for degrading three- and four-ring PAHs, consistent with the catabolic spectrum of members belonging to the obligate PAH-degrading genera *Cycloclasticus* (3) and *Neptunomonas* (4). Strain MCTG13d is a strictly aerobic and motile, rod-shaped bacterium that is associated with various species of marine diatoms and dinoflagellates found in different seas and oceans worldwide (1; T. Gutierrez, unpublished results).

Here, we report the genome sequence of *Porticoccus hydrocarbonoclasticus* strain MCTG13d. Genomic DNA was isolated, and the sequence was generated at the Department of Energy (DOE) Joint Genome Institute (JGI; Walnut Creek, CA, USA) using Pacific Biosciences (PacBio) technology. A PacBio SMRTbellTM library was constructed and sequenced on the PacBio RS platform, which generated 189,901 filtered subreads totaling 628.8 Mbp. All general aspects of library construction and sequencing performed at the JGI can be found at http://www.jgi.doe.gov. The raw reads were assembled using HGAP version 2.1.1 (5). The final draft assembly produced 1 scaffold containing 1 contig totaling 2.5 Mbp and input read coverage of 291.1×.

Project information is available in the Genomes OnLine Database (6). Genes were identified using Prodigal (7), followed by a round of manual curation using GenePRIMP (8) as part of the JGI’s microbial annotation pipeline (9). The predicted coding sequences were translated and used to search the National Center for Biotechnology Information (NCBI) nonredundant, UniProt, TIGRFam, Pfam, KEGG, COG, and InterPro databases. The tRNAscanSE tool (10) was used to find tRNA genes, whereas rRNA genes were found by searches against models of the rRNA genes built from SILVA (11). Other noncoding RNAs such as the RNA components of the protein secretion complex and the RNase P were identified by searching the genome for the corresponding Rfam profiles using Infernal (http://infernal.janelia.org). Additional gene prediction analysis and manual functional annotation was performed within the Integrated Microbial Genomes Expert Review (IMG ER) platform (http://img.jgi.doe.gov) developed by the JGI (12).

The complete genome sequence length was 2,474,654 bp with a G+C content of 53.1%. The genome contains 2,385 genes (2,340 protein-coding genes) with functional predictions for 2,021 of them. A total of 45 RNA genes were detected. Other genes, characteristic for the genus, are given in the IMG database (12). This genome sequence is expected to provide great insights into the unusual life style of this organism.

### Nucleotide sequence accession number.

The draft genome sequence of *P. hydrocarbonoclasticus* strain MCTG13d obtained in this study was deposited in GenBank as part of BioProject number PRJNA224116, with individual genome sequences submitted as whole-genome shotgun projects under the accession number JQMM00000000.
